# Prevalence and antimicrobial resistance profiles of extended-spectrum beta-lactamase-producing *Escherichia coli* in East Tennessee dairy farms

**DOI:** 10.3389/fvets.2023.1260433

**Published:** 2023-12-18

**Authors:** Benti D. Gelalcha, Aga E. Gelgie, Oudessa Kerro Dego

**Affiliations:** Department of Animal Science, The University of Tennessee, Knoxville, TN, United States

**Keywords:** ESBL *Escherichia coli*, prevalence, dairy farm, multidrug resistance, antimicrobial susceptibility, antimicrobial resistance, dairy cattle, calves

## Abstract

**Introduction:**

The extended-spectrum beta-lactamase (ESBL)-producing *Enterobacteriaceae,* such as *Escherichia coli*, are emerging as a serious threat to global health due to their rapid spread and their multidrug-resistant (MDR) phenotypes. However, limited information is available regarding the prevalence and antimicrobial resistance (AMR) profile of ESBL-*E. coli* in the United States dairy farms. This study aimed to determine the prevalence and AMR pattern of ESBL-*E. coli* in East Tennessee dairy cattle farms.

**Methods:**

Rectal fecal samples from dairy cattle (*n* = 508) and manure (*n* = 30), water (*n* = 19), and feed samples (*n* = 15) were collected from 14 farms. The presumptive *E. coli* was isolated on CHROMagar™ ESBL and confirmed by matrix-assisted laser desorption/ionization-time of flight mass spectrometry (MALDI-TOF MS). Antimicrobial susceptibility testing was performed on the ESBL-*E. coli* isolates.

**Results and discussion:**

From 572 fecal and farm environmental samples, a total of 233 (41%, *n* = 572) ESBL-*E. coli* were identified. The prevalence of fecal ESBL-*E. coli* was 47.5% (95% CI: 46.2–49.2). The within-farm prevalence of ESBL-*E. coli* ranged from 8 to 100%. Recent treatment history with third-generation cephalosporins (3GC), cow parity ≥3, and calves were the independent risk factors associated (*P* < 0.05) with fecal carriage of ESBL-*E. coli*. Overall, 99.6% (*n* = 231) ESBL-*E. coli* tested were phenotypically resistant to at least one of the 14 antimicrobial agents tested. The most common AMR phenotypes were against beta-lactam antibiotics, ampicillin (99.1%; *n* = 231 isolates), and ceftriaxone (98.7%, *n* = 231). Most ESBL-*E. coli* isolates (94.4%) were MDR (resistance to ≥3 antimicrobial classes), of which 42.6% showed co-resistance to at least six classes of antimicrobials. ESBL-*E. coli* isolates with concurrent resistance to ceftriaxone, ampicillin, streptomycin, tetracycline, sulfisoxazole, and chloramphenicol are widespread and detected in all the farms. The detection of MDR ESBL-*E. coli* suggests that dairy cattle can be a reservoir for these bacteria, highlighting the associated public health risk.

## 1 Introduction

Antimicrobial resistance (AMR) is recognized as one of the top five global public health threats of this century (1). Of great concern are extended-spectrum beta-lactamase (ESBL)-producing *Enterobacteriaceae,* including *Escherichia coli,* due to their rising prevalence in food-producing animals (2, 3). Some studies identified the use of β-lactam antibiotics, especially the third generation cephalosporins (3GC) in dairy cattle farms, as a major risk factor for the rise of ESBL-producing *Enterobacteriaceae* (4, 5).

In the United States, β-lactam antibiotics such as 3GC (e.g., ceftiofur), first-generation cephalosporins (e.g., cephapirin), and penicillin are the top three most frequently used antibiotics in dairy cattle (6, 7). These antibiotics are mainly used to treat or prevent mastitis, metritis, endometritis, lameness, pneumonia or bovine respiratory disease complex, and neonatal calf diarrhea (8–10). According to FDA’s 2019 report, from 29,830 kg of cephalosporins sold and approved for use in food-producing animals, the vast majority (81%) was distributed to cattle production (11). Similarly, Nora et al. (12) also reported that dairy cattle use the largest amount of cephalosporins and penicillin, both of which are β-lactam antibiotics.

According to the WHO’s risk-based classification systems, third-generation cephalosporins are categorized as a ‘critically important class of antibiotics (CIAs) (13). They are used as antimicrobial drugs of choice for the treatment of severe infections caused by *E. coli* and *Salmonella* in humans (14, 15). The use of the same generation of cephalosporins in dairy cattle farms and human health settings may lead to cross-resistance to similar cephalosporins used for the treatment of human infections or vice versa if resistant bacteria transfer from carrier animals to humans through direct contact or indirectly through the food chain or environmental sources (16, 17).

In *Enterobacteriaceae*, resistance to 3GC is mainly mediated by the production of ESBL, a group of enzymes that hydrolyze the β-lactam ring of the 3GCs (18). ESBL-producing *Enterobacteriaceae* can be multidrug-resistant (MDR) and display resistance to other classes of antibiotics such as tetracycline, aminoglycosides, fluoroquinolones, sulfonamides, macrolide, and phenicols (19–24). *E. coli,* the most frequent colonizer of the gastrointestinal tracts of cattle (25), are frequently exposed to β-lactam antibiotics that exert selection pressure, and certain strains of *E. coli* can cause severe infections in humans (17, 25–29). Recent studies in the United States (16, 30–34) increasingly reported the emergence of ESBL-producing *E. coli*, in dairy cattle since its first report in Ohio in 2010 (32), posing a significant threat to both animal and human health.

Similarly, reports from the United States Centers for Disease Control and Prevention (CDC) indicated a continuous rise in human infections caused by community-associated ESBL-producing *Enterobacteriaceae* (35). The CDC report revealed a yearly rise of approximately 9% in hospitalizations and an increase in deaths attributed to ESBL-producing bacteria. There is speculation that dairy farms may serve as reservoirs of ESBL-producing human pathogens because of the frequent use of 3GC (29, 36–38). The CDC has not directly implicated dairy farms as a source of ESBL-producing *Enterobacteriaceae* infection. However, the frequent use of 3GCs and other β-lactam antibiotics in dairy farms may play a role in the overall increase of ESBL-producing *Enterobacteriaceae*.

Previous studies on the United States dairy cattle farms showed an increasing trend in the occurrence of ESBL-producing *E. coli* (4, 30, 32, 33, 37, 39). A recent review of available literature on United States dairy cattle farms and recent global reviews on the status of ESBL-producing *Enterobacteriaceae* in cattle indicated that there is limited information on the status of ESBL-producing *Enterobacteriaceae,* including ESBL-producing *E. coli* in the United States dairy farms (16, 40). The prevalence of ESBL-producing *E. coli* and factors affecting its occurrence in dairy farms are poorly understood. Understanding the status (prevalence, risk factors and resistance profile) of ESBL-producing *E. coli* is crucial to informing the associated public health risks and devising effective control measures. The overall objective of this study was to determine the prevalence and antimicrobial susceptibility patterns of ESBL-producing *E. coli,* in East Tennessee dairy cattle and farm environmental samples.

## 2 Materials and methods

### 2.1 Study design and sample size

This study was approved by the University of Tennessee’s Institutional Animal Care and Use Committee (IACUC) Registration Number: 2782–0720. The study farms were conventional (non-organic) dairy farms in East Tennessee. A cross-sectional study was conducted in 14 dairy farms across eight counties in East Tennessee.

East Tennessee was selected due to its proximity to the University of Tennessee, and most of the dairy farms (59%, *n* = 183) in Tennessee are found in this part of the State. Dairy farms were randomly selected (using a random number generator) from a sampling frame of 108 dairy farms found at the beginning of this study in East Tennessee. Since some farmers may not be interested in the study, the first 60 dairy farms were randomly selected. Dairy farmers on the list were then contacted one at a time by phone and asked whether they were willing to participate in the study. When the first selected farm refused to participate or could not be contacted, the next farms on the list were contacted and asked for their willingness to participate in the study. In the end, of the contacted dairy farms (*n* = 50), only 14 agreed to participate in the study.

To estimate the sample size, 50% expected prevalence of ESBL-*E. coli* at an animal level, 0.05 desired precision at a 95% confidence interval was used. Accordingly, the minimum required sample size was 384. To account for the possible clustering effect (design effect), the minimum sample size (*n* = 384) was multiplied by 1.34 (design effect) (4, 41). Accordingly, 507 dairy cattle were sampled (9–74 animals per farm). The herd size of the farms ranges from 14 to 1700 dairy cattle. Convenience sampling was used to select an individual animal in the herd. On the day of the farm visits, a brief questionnaire survey was conducted with a dairy farmer (producer) before sample collection. The questions included farm-related information such as herd size, predominant breed of cows, major disease problems in the farm, common antibiotics used (for therapeutic and prophylactic purposes), use of a blanket dry cow therapy (BDCT) versus selective dry cow therapy (SDCT), management of waste milk (milk from antibiotic-treated cows), use of medicated milk replacer, management of manure, and type of farm (open or closed), if open the recent introduction of animals from other herds. Individual sampled animal-related information such as age, breed, parity, physiological status (dry/lactating), recent treatment history, and use of ceftiofur within the last 6 months before sample collection.

### 2.2 Sample collection

Following the recruitment of the farms, one-time visits were made to each farm between August 2020 and July 2021 for survey data and sample collections. Fresh rectal fecal samples were collected from individual cows and calves using sterile disposable rectal long-arm gloves. Farm environmental samples such as manure (pooled from different pen surfaces and slurry), feed (pooled feed), and water (from troughs) were collected from the study farms. Approximately 100 g of individual cow and 20 g of individual calf rectal feces were collected into 50 mL sterile falcon tubes (Thermo Fischer Scientific, Waltham, MA, United States) and labeled with individual animal ID. Similarly, about 200 g of manure and feed samples and 50 mL of water from water troughs were collected into 50 mL sterile tubes. The samples were immediately placed on ice in an icebox, transported to the Lab, and processed in less than 24 h of collection.

The study recruited 14 dairy farms (designated A-N) across eight counties in East Tennessee (Table 1). A total of 424 dairy cows (average parity: 2.3 lactations, range: 1–8 lactations; average age: 3.9 years, range: 2–13 years) and 84 calves (average age: 2.7 months old, range: 12 days to 8 months). Pooled manure (*n* = 30), pooled water samples (*n* = 19) from water troughs, and polled feed samples (*n* = 15) were collected from the farms (Table 1). Two samples (from farm N), one collected from a lagoon and the other one collected from runoff from the farm to the environment, were included as manure. A feed sample was not collected from one of the farms (Farm M) as the farmer was less comfortable. One of the two collected feed samples in farm G was a liquid whey fed to cattle.

**Table 1:** Farm location and samples collected.

County	Farm	Fecal samples collected		Manure	Water	Feed	Total
		Cows	Calves				
1	A	14	–	2	1	1	18
	B	9	–	1	1	1	12
2	C	63	–	2	1	1	67
	D	30	–	2	1	1	34
3	E	13	–	2	1	1	17
	F	30	10	2	1	1	44
4	G	25	–	1	1	2	29
5	H	26	–	1	1	1	29
6	I	20	10	2	1	1	34
	L	41	9	2	2	1	55
	N	55	19	7	4	2	87
7	J	20	5	2	1	1	29
	K	30	15	2	1	1	49
8	M	48	16	2	2	-	68
All farms		424	84	30	19	15	572

### 2.3 Laboratory analysis of collected samples

About 10 g of individual animal rectal feces, feed, and manure samples were combined with 90 mL of Tryptic soy broth (TSB-PO_4_, MG Scientific, Pleasant Prairie, WI, United States) in a Whirl-Pak bag (Whirl-Pak, Pleasant Prairie, WI, United States) and massaged by hand for 15–20 s to mix the samples with the TSB-PO4. For water samples, 20 mL of sample was mixed with 80 mL TSB-PO_4_. The homogenized sample was placed at room temperature for about 2 h. Then, 50 μL of the homogenized sample was spirally platted on CHROMagar ESBL plates (DRG International Inc., Springfield, NJ, United States) and incubated at 37°C for 24 h to isolate ESBL-*E. coli*. Two to three presumptive ESBL-*E. coli* (dark pink to reddish) colonies were subcultured onto a new CHROMagar ESBL plate and incubated for another 24 h at 37°C. Well-isolated pure colonies were transferred to 1 mL Luria-Bertani broth (LBB) (Thermo Fischer Scientific) into a sterile 2 mL 96 well-serum block and incubated at 37°C for 24 h. A 0.5 mL of the culture was transferred to a new 96-well serum block, mixed with an equal volume of sterile 80% glycerol, and stored at −80°C for further analysis.

### 2.4 Sample preparation and MALDI-TOF MS based *Escherichia coli* identification

Presumptive *E. coli* isolates stored at −80°C were thawed and plated on CHROMagar ESBL plates ESBL plates and incubated at 37°C for about 18 h. A single colony of *E. coli* was picked and subcultured on blood agar (Thermo Fischer Scientific) at 37°C for about 18 h. Subsequently*, E. coli* was identified using matrix-assisted laser desorption ionization time-of-flight mass spectrometry (MALDI-TOF MS) as described by the manufacturer (Bruker Daltonics, Billerica, MA, United States) at the University of Tennessee, College of Veterinary Medicine, Diagnostic Bacteriology and Mycology Lab. The samples were prepared using a formic acid (FA) extraction method (42) and as described in detail in our previous publication (43).

### 2.5 Antimicrobial susceptibility testing

Antimicrobial susceptibility testing (AST) was performed on MALDI-TOF MS confirmed *E. coli* isolates against 14 antimicrobials representing ten classes of antimicrobials using the broth microdilution method. Commercially available 96-well microtiter plates containing the 14 antimicrobial panels (Sensitire™ CMV4AGNF) (Thermo Fisher Scientific) described in our previous publication were used (43). The minimum inhibitory concentrations (MIC) of the 14 antimicrobials were determined following the manufacturer’s recommended protocol and following CLSI M100 Clinical and Laboratory Standards Institute guidelines (CLSI M100: *Enterobacteriaceae*) (44) and summarized (Supplementary Table S1). *E. coli* ATCC 25922, *Staphylococcus aureus* ATCC 29213, *Streptococcus pneumoniae* ATCC 49619, *E. coli* ATCC 25922, and *Pseudomonas aeruginosa* ATCC 27853 were used as quality control strains.

The results were reported as susceptible (S), Intermediate(I), resistant (R) along with the MIC values, the lowest concentration (μg/mL) of an antibiotic that completely inhibits visible growth of the tested isolates, using CLSI M100 guidelines for interpretation.

An MIC breakpoint was not available on CLSI M100 guidelines for streptomycin and, thus, National Antimicrobial Resistance Monitoring System (NARMS) interpretive criteria (breakpoints) for *E. coli* antimicrobial susceptibility testing^1^ was used. Accordingly, an MIC ≥32 μg/mL was defined as resistant to streptomycin. For sulfisoxazole, CLSI defines an MIC ≤256 μg/mL as susceptible and an MIC ≥512 mL as resistant, and there are no breakpoints for intermediate resistance. Thus, *E. coli* isolates that have an MIC ≥256 μg/mL were reported as resistant to sulfisoxazole in this study. In addition, for analyses, a few *E. coli* isolates that displayed intermediate resistance, mostly to ciprofloxacin, were recategorized as resistant. Multidrug resistance (MDR) was defined as acquired resistance to at least one antimicrobial agent in three or more antimicrobial classes (45).

### 2.6 Statistical data analyses

Raw data was entered in Microsoft Excel for Windows (2010, Microsoft Corp., Redmond, WA). Data was imported to SPSS for Windows and analyzed using IBM SPSS Statistics for Windows Version 27.0. (IBM Corp, Armonk, NY, United States). For all statistical analyses, the unit of analysis was the sample obtained from a given source (cow, calf, manure, water, and feed), the farms, and the bacterial isolates. Descriptive statistics were done to summarize the animal and farm-level prevalence of ESBL-*E. coli* and the prevalence of the bacteria from different sources (fecal, water, feed, and manure samples) of the farms. The individual animal was classified as ESBL-positive when ESBL-producing *E. coli* isolates were identified phenotypically on the CHROMagar ESBL™ plate and confirmed by MALDI-TOF MS (46, 47) in the feces of the dairy cow or calf and the other samples.

A farm (herd) was classified as ESBL-positive when ESBL-producing *E. coli* was detected from at least one animal. Descriptive statistics were used to calculate the prevalence of ESBL-producing *E. coli* as the number of samples tested positive for ESBL-producing *E. coli* divided by the total number of samples tested for each sample category. Sampling weight was used to calculate the within-herd and individual animal level prevalence to account for the unequal probability of sampling employed during the sample collection. The weighted sample for a specific farm was determined by dividing the actual number of animals sampled on the farm by the herd size of that farm. The reciprocal of this quotient was then taken as the weighted sample for the farm. To calculate the overall weighted prevalence, the unweighted prevalence of ESBL-producing *E. coli* in each of the 14 herds (farms) was multiplied by the corresponding herd proportion. These products were then summed up, and the result was divided by 100. A univariate and multivariable mixed-effects logistic regression model, which accounts for the clustering effect of cattle or isolates within farms, was used in the analysis. A mixed effects logistic regression analysis (weighted) was performed to evaluate the association between selected predictors (farm or animal level data) and the odds of ESBL-producing *E. coli* fecal carriage (dependent variable) in cattle.

In addition, the multidrug resistance (MDR) status of each bacterial isolate obtained from the study was treated as a dichotomous outcome variable (MDR vs. non-MDR), and their possible variation between sample sources was evaluated. The MDR isolates were further dichotomized (whether they were resistant to at least six classes of antibiotics or not) and assessed if there was a variation between the sample sources. A Pearson *χ*^2^ or Fisher exact test (as appropriate) was used to test the association between different categorical variables (ignoring clustering) when the mixed effect model fails to converge. A value of *p* = 0.05 was used for all statistical analyses to determine the significance level.

## 3 Results

### 3.1 Questionnaire survey results

The questionnaire survey showed that half of the farms practice closed production systems where replacement heifers originated from the same farm, whereas the other half buy replacement heifers/cows from outside sources in addition to raising their own. Thirteen of the fourteen participating dairy farms use cow manure as fertilizer, mainly on pasture, and one farm (Farm G) uses manure for biogas production. Most of the farms (71%) discard waste milk from cows on antibiotic treatment (dump down the drain or dump on the pasture field), whereas the remaining four farms (Farm C, F, G, and I) feed it to calves. A blanket dry cow treatment program was routinely practiced in all the farms except three (B, F, and N), which used selective dry cow therapy and teat sealant. The most frequently used antibiotics for dry cow therapy were Spectromast^®^ DC (ceftiofur hydrochloride), toMORROW (cephapirin benzathine), and Quartermaster (procaine penicillin G and dihydrostreptomycin). Eleven farms predominantly have Holstein-breed cows, whereas Jersey cows dominate the remaining three. The herd size of the 14 farms ranged from 14 to 1700 cattle, with a median herd size of 750 animals.

All farmers mentioned mastitis as the most frequent herd health problem, followed by hoof problems. Beta-lactam antibiotics (penicillin and cephalosporins) were the most common antibiotics used in all farms. All study farms frequently use different generations and formulations of cephalosporins. Major disease problems and frequently used antibiotics in each farm are listed in Table 2.

**Table 2:** Common diseases and antibiotics used in each dairy farm.

Farm	Common diseases on the farms					Commonly used antibiotics^a^
	Mastitis	Hoof problem	Metritis/endometritis	Resp. disease	GIT disease	
A	Yes	Yes	No	No	No	Ceftiofur, pirsue, and quartermaster
B	Yes	No	No	Yes	No	Tetracycline, pirsue, toDAY, ToMORROW
C	Yes	Yes	No	Yes	No	ToMORROW and ceftiofur
D	Yes	Yes	Yes	No	No	Ceftiofur, penicillin, and quartermaster
E	Yes	No	No	No	No	ToDAY and ToMORROW
F	Yes	Yes	No	No	No	Ceftiofur and quartermaster
G	Yes	Yes	No	No	No	Tetracycline, Penicillin, ToMORROW, quartermaster
H	Yes	No	No	Yes	No	Ceftiofur, ampicillin
I	Yes	Yes	No	No	No	Pirsue, ToDAY, and ToMORROW
J	Yes	Yes	No	Yes	Yes	Ceftiofur, penicillin G, ampicillin, ToDAY, pirsue, and ToMORROW
K	Yes	Yes	Yes	Yes	Yes	Ceftiofur, Penicillin G, ampicillin, quartermaster, tulathromycin, and ToMORROW
L	Yes	Yes	No	No	No	Ceftiofur, ToMORROW, and quartermaster
M	Yes	Yes	Yes	Yes	No	Ceftiofur, penicillin G, and tetracycline
N	Yes	Yes	Yes	Yes	Yes	Ceftiofur, penicillin G, tetracycline, and tulathromycin

^a^toMORROW, cephapirin benzathine; toDAY, cephapirin sodium; Pirsue, pirlimycin hydrochloride; Quartermaster, Procaine penicillin G and Dihydrostreptomycin sulfate; Endometr, endometritis; Resp, respiratory; GIT, gastrointestinal.

### 3.2 Microbiological result

According to CHROMagar™ ESBL result, a total of 306 presumptive ESBL-*E. coli* were isolated from rectal feces, manure, water, and feed samples. Out of 306 presumptive ESBL-*E. coli* isolates, 233 (76%) were confirmed to be *E. coli by* MALDI-TOF MS. The remaining bacteria include *Citrobacter sedlakii* (*n* = 69 isolates), *Citrobacter freundii* (*n* = 2), and *Acinetobacter baumanii* and *Enterococcus faecalis*. One each. Except for AST, which was conducted on 231 isolates, all statistical analyses and inferences were based on the 233 *E. coli* isolates. Most of the *E. coli* isolates (90%, *n* = 233) were isolated and identified from rectal fecal samples, whereas the remaining (10%) were from farm environmental samples (manure, water, and feed samples).

### 3.3 Prevalence of ESBL-producing *Escherichia coli* isolates

The weighted prevalence of fecal ESBL-producing *E. coli* in dairy cattle was 47.5% (95% CI: 46.2–49.2). ESBL-producing *E. coli* was detected from all farms. The prevalence varies widely between farms, with the highest within-herd weighted prevalence of 100% from farm A, whereas the lowest prevalence of 8% was from two farms (farms E and N). ESBL-producing *E. coli* was isolated from fecal samples of all fourteen farms, resulting in a herd-level prevalence of 100%. The herd size of the farms, the number of animals tested from each farm, the number of animals that tested positive and within-herd weighted prevalence, and the 95% confidence interval (95% CI) of the estimate for each farm are presented in Table 3.

**Table 3:** Within-herd prevalence of ESBL *Escherichia coli* across fourteen dairy farms.

Farm	Herd size^a^	Sample size (*n*)^b^	Unweighted frequency (prevalence %)^c^	95% CI unweighted prevalence	Weighted frequency and c^d^	95% CI of the weighted prevalence
A	100	14	14 (100)	76.8–100	100 (100)	96.4–100
B	14	9	3 (33.3)	7.4–70	5 (35.7)	12.8–64.9
C	750	63	35 (55.6)	42.4–68	417 (55.6)	52.0–59.2
D	300	30	3 (10)	2.2–26.5	30 (10)	6.8–13.9
E	50	13	1 (7.7)	0.2–36	4 (8)	2.2–19.2
F	110	40	27 (67.5)	50.8–81.4	74 (67.3)	57.7–75.9
G	170	25	6 (24)	9.4–45.1	41 (24.1)	17.9–31.3
H	35	26	22 (84.6)	65.1–95.6	30 (85.7)	69.7–95.2
I	61	30	4 (13.3)	3.8–30.7	8 (13.1)	5.8–24.2
J	60	25	18 (72)	50.6–87.9	43 (71.7)	58.6–82.5
K	1700	45	28 (62.2)	46.5–76.2	1,058 (62.2)	59.9–64.5
L	250	50	21 (42)	28.2–56.8	105 (42)	35.6–48.4
M	450	64	21 (32.8)	3.0–16.8	148 (32.9)	28.6–37.4
N	350	74	6 (8.1)	3.0–16.8	28 (8)	5.4–11.4
Total	4,400	508	209 (41.1%)	36.8–45.6	2090 (47.5%)	46.2–49.2

^a^Herd size: the total number of animals on each farm; ^b^The actual number of animals sampled from each farm; ^c^The number and proportion of animals from which ESBL *Escherichia coli* was isolated; ^d^Frequency of the weighted sample size and the corresponding prevalence in the herd.

**Figure 1:**
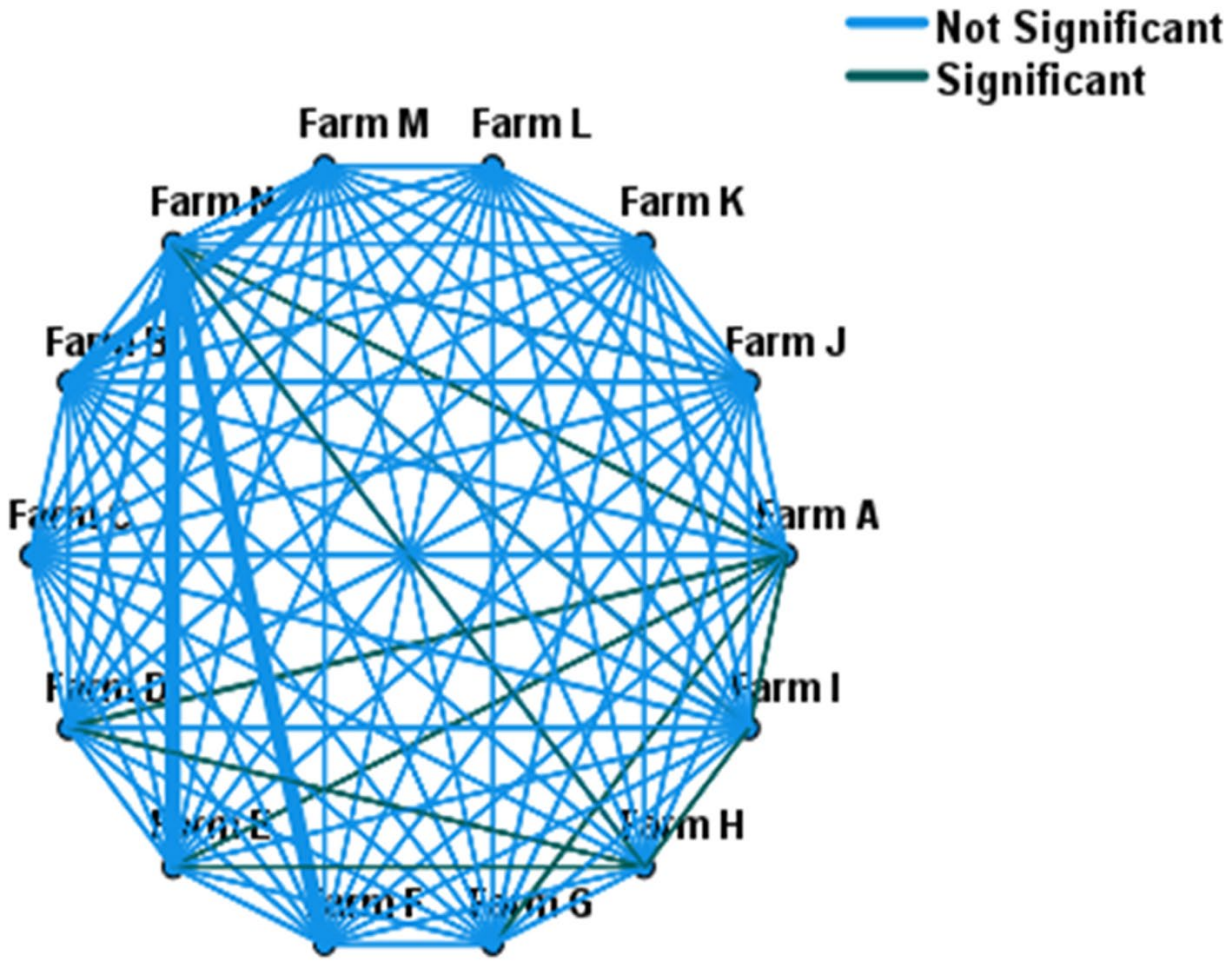
Pairwise comparison of within-herd fecal ESBL-E. coli prevalence across 14 farms. Farms connected with green lines (—) showed statistically significant variation in their within-herd prevalence, whereas no difference for those farms connected with light blue lines (—).

The Chi-square test of independence was performed (without accounting for within farm clustering effect of the isolates) to test if there was a statistically significant difference in the weighted prevalence of ESBL-producing *E. coli* between farms. The test showed an overall statistically significant variation (*χ*^2^ = 21; *p* < 0.001) of within-herd prevalence of ESBL-producing *E. coli.* Similarly, a mixed-effect logistic regression (controlling the random effect of the farm) showed an overall significant variation in the fecal ESBL-producing *E. coli* prevalence between farms (*p* < 0.001). A comparison of the within-herd prevalence of fecal ESBL-producing *E. coli* between individual farms is shown in Figure 1.

The weighted prevalence of fecal ESBL-producing *E. coli* was 44 and 61.7% in cows and calves, respectively. Calves were twice [OR = 2.12 (95% CI: 1.78–2.53), *p* < 0.001] as likely to carry ESBL-*E. coli* in their feces compared to cows using mixed effect logistic regression analysis with farms as a random variable and herd structure (calf vs. cow) as the sole fixed variable (Table 4).

**Table 4:** Comparison of prevalence of fecal ESBL *Escherichia coli* in dairy cattle.

Herd structure	Unweighted sample size	Weighted size	Weighted frequency and [(prevalence), 95% CI]	OR (95% CI)	*p*-value
Cows	424	3,526	1,551 [(44%), 42.3–45.6]	Ref	
Calves	84	874	539 [(61.7%), 58.4–64]	2.12 (1.78–2.53)	<0.001
Total	508	4,400	2090 [(47.5%), 46.2–49.2]	NA	

ESBL-producing *E. coli* was recovered from over a quarter of all sample types (26%). The highest proportion of ESBL-producing *E. coli* was obtained from pooled manure samples (46.7%), whereas the least (26.3%) was from water samples (Supplementary Table S2).

### 3.4 Factors affecting fecal ESBL-producing *Escherichia coli* prevalence in dairy cattle

After matching the farm-level survey data with the findings of the MALDI-TOF MS results from individual animals, a mixed-effect logistic regression analysis was carried out to determine the association between the variables collected during the survey and the probability of isolating ESBL-producing *E. coli* in samples. The farm-level collected data include herd size, farm type (closed vs. open), predominant breed in the farm, use of blanket dry cow therapy (BDCT) vs. selective dry cow therapy (SDCT), types of antibiotics used for DCT, presence of treatment ward in the farm, management of waste milk from treated cows (discard vs. fed to calves). None of these farm-level variables were associated with the detection of ESBL-producing *E. coli* in fecal samples, in univariate and multivariable analysis (*p* > 0.05), and, thus, were not included in this report.

The relationship between individual animal-related surveys such as the recent (up to 6 months prior to sampling collection) use of beta-lactam antibiotics such as ceftiofur, parity, lactation status of the cows (lactating vs. dry or non-lactating), age (of cows), and the breed was assessed for possible association with fecal ESBL-producing *E. coli* carriage. First, a univariate analysis was conducted to test the association between independent variables and the likelihood of isolating ESBL-producing *E. coli* in fecal samples. Then, only those variables with a *p* ≤ 0.2 in univariate analyses were used in a multivariable analysis controlling the random effect of the farm. Overall, recent treatment with ceftiofur, cow parity, and the lactation status of the cows was independently associated (*p* < 0.001) with the prevalence of fecal ESBL-producing *E. coli*. The odds of ESBL-producing *E. coli* fecal carriage in cows recently treated with ceftiofur was higher than [OR_adj_ = 1.35 (95% CI: 1.1–1.65), *p* = 0.004] those which did not receive the treatment. Lactating cows were more than three times [OR_adj_ = 3.42 (95% CI: 2.79–4.10), *p* < 0.001] more likely to carry ESBL-*E. coli* in their feces compared to dry cows.

Age and parity of the cows were highly correlated (*p* < 0.001; *R* = 0.73) since parity increases with age. Thus, the age of the cows was not included in the analysis, and parity was retained in the model. Odds of retrieving ESBL-producing *E. coli* was 33% higher [OR_adj_ = 1.33 (95% CI: 1.13–1.63), *p* = 0.01] in cows with parity greater than two compared to those cows with parity less than or equal to two (Table 5).

**Table 5:** Association between cow-level characteristics and weighted fecal ESBL *Escherichia coli* prevalence.

Variable		Prevalence (frequency)	Multivariable analyses	
Name	Category		Adjusted OR (95% CI)	*p*-value
Ceftiofur*Treated	No	15.4% (254/1641)		
	Yes	20.5% (230/1124)	1.35 (1.1–1.65)	0.004
*Reproductive status*				
	Dry	22.2% (180/812)		
	Lactating	50.5% (1,371/2714)	3.42 (2.79–4.10)	<0.001
Cow parity	≤2	39.4% (840/2130)		
	≥3	50.8% (706/1391)	1.33 (1.13–1.56)	<0.001

^*^The data related to treatment with ceftiofur pertains only to cows (does not include claves).

### 3.5 Antibiotic resistance profile of ESBL-producing *Escherichia coli* isolates from all sample type

Overall, 230 (99.6%) of 231 *E. coli* tested for phenotypic resistance were resistant to at least one of the 14 antimicrobial agents tested (Table 6). One isolate was susceptible to all tested antimicrobials. The most common resistance phenotypes were against beta-lactam antibiotics, ampicillin (99.1%; *n* = 231), and ceftriaxone (98.7%, *n* = 231). Only three isolates showed susceptibility to ceftriaxone; two of them were recovered from cows. After ceftriaxone and ampicillin, the isolates showed the highest resistance to tetracycline (80.1%), sulfisoxazole (60.2%), streptomycin (55%), and chloramphenicol (46.8%).

**Table 6:** Number and percentage of resistance of ESBL *Escherichia coli* isolates (*N* = 231) obtained from various sources.

Antibiotic	Sources of *E. coli* isolates and proportion of resistance to the specific antibacterial agent					Prevalence of resistant isolates to specific antibiotic
	Cows (*N* = 180)^a^	Calves (*N* = 27)	Manure (*N* = 14)	Feed (*N* = 5)	Water (*N* = 5)	
	*n* (% Resistant)^b^	*n* (% Resistant)	*n* (% Resistant)	*n* (% Resistant)	*n* (% Resistant)	*n* (% Resistant)
*AUG2*	8 (4.4)	11 (40.7)	0 (0)	1 (20)	0 (0)	20 (8.7)
*AMP*	178 (98.9)	27 (100)	14 (100)	5 (100)	5 (100)	229 (99.1)
*AZI*	25 (13.9)	4 (14.8)	0 (0)	2 (40)	0 (0)	31 (13.4)
*FOX*	11 (6.1)	11 (40.7)	1 (7.1)	0 (0)	0 (0)	23 (10)
*AXO*	178 (98.3)	26 (96.3)	14 (100)	5 (100)	5 (100)	228 (98.7)
*CHL*	83 (46.1)	12 (44.4)	7 (50)	3 (60)	3 (60)	108 (46.8)
*GEN*	9 (5)	12 (44.4)	1 (7.1)	0 (0)	0 (0)	22 (9.5)
*MERO*	0 (0)	0 (0)	0 (0)	0 (0)	0 (0)	0 (0)
*CIP*	36 (20)	0 (0)	2 (14.3)	3 (60)	2 (40)	43 (18.6)
*NAL*	16 (8.9)	4 (14.8)	0 (0)	1 (20)	0 (0)	21 (9.1)
*STR*	85 (47.2)	25 (92.6)	9 (64.3)	4 (80)	4 (80)	127 (55)
*FIS*	107 (59.4)	20 (74.1)	9 (64.3)	1 (20)	2 (40)	139 (60.2)
*TET*	138 (76.7)	25 (92.6)	14 (100)	4 (80)	4 (80)	185 (80.1)
STX	44 (24.4)	12 (44.4)	3 (21.4)	1 (20)	1 (20)	61 (26.4)

^a^*N*, total number of isolates obtained from a given source; ^b^*n*, number of isolates resistant to a specific antimicrobial agent; proportion: the percentage of isolates resistant to a particular antimicrobial in the given sample (*n*/*N*). AXO, ceftriaxone; FOX, cefoxitin; AUG2, amoxicillin-clavulanic acid; AMP, ampicillin; MERO, meropenem, AZI, azithromycin; CIP, ciprofloxacin; NAL, nalidixic acid; CHL, chloramphenicol; GEN, gentamicin; STR, streptomycin; TET, tetracycline; FIS, sulfisoxazole; STX, trimethoprim-sulfamethoxazole.

Among the critically important classes of antibiotics, the higher level of resistance after ceftriaxone was to streptomycin (55%), followed by ciprofloxacin (18.6%) and azithromycin (13.4%). The prevalence of resistance to gentamycin and nalidixic acid, the other critically important antimicrobials tested in this study, were 10 and 9%, respectively.

The resistance level to other β-lactam antibiotics was 10% for cefoxitin and 9.2% for amoxicillin-clavulanic acid. All ESBL-producing *E. coli* isolates in this study were susceptible to meropenem.

### 3.6 Multidrug resistance profiles of ESBL-producing *Escherichia coli* isolates

Most of the ESBL-producing *E. coli* isolates (94.4%; 218/231) displayed multidrug resistance (MDR) phenotypes (acquired resistance to at least one agent in three or more antimicrobial classes) (45). All isolates recovered from manure (*n* = 5) and water samples (*n* = 5) showed MDR phenotypes. Most of the isolated from calves (96.3%; *n* = 27), cows (93.9%; *n* = 180), and feed (80%; *n* = 4) were MDR. Among the 14 farms, all isolates obtained from 10 of the farms were MRD. From 218 MDR isolates, 42.6% showed concurrent resistance to at least one antibiotic in six classes of antibiotics. ESBL-producing *E. coli* isolates that showed concurrent resistance to ≥6 antimicrobial classes were obtained from all sample sources (Supplementary Table S3).

### 3.7 Distribution of ESBL-producing *Escherichia coli* resistant to multiple antimicrobial agents

Overall, resistance to multiple classes of antibiotics is widespread across the study farms (Table 7). The most common (52.8%, 122/231) and widespread multidrug resistance pattern was concurrent resistance to ceftriaxone, ampicillin, streptomycin, and tetracycline, detected in all farms and sample types. Similarly, simultaneous resistance to ceftriaxone, ampicillin, sulfisoxazole, and tetracycline was frequent (48.5%, 112/231) and detected in all farms and sample types. Concurrent resistance to at least one antimicrobial agent in all critically important classes of antibiotics (ceftriaxone, streptomycin, azithromycin, and ciprofloxacin) was relatively less frequent (6.5%, 15/231) and limited in the scope of spread among farms (detected in only three farms). Fourteen of fifteen *E. coli* isolates with this resistance pattern were retrieved from cow fecal samples. Seventeen (7.4%) of *E. coli* isolates were co-resistant to all beta-lactam antibiotics tested in this study (ceftriaxone, ampicillin, cefoxitin, and amoxicillin-clavulanic acid). *E. coli* isolates with this co-resistance phenotype were detected in six farms, and all of them were isolated from fecal samples except one recovered from a feed sample.

**Table 7:** Distribution of concurrent resistance of ESBL *Escherichia coli* to multiple classes of antibiotics across farms and sample sources.

Concurrent resistance pattern	Sources and number of isolates				Prevalence and No. of farms with the resistance pattern	
	Feces (*N* = 207)	Manure (*N* = 14)	Water (*N* = 5)	Feed (*N* = 5)	*n* (Prevalence)^a^	No. of farms^b^
AXO-AMP-STR-TET	105	9	4	4	122 (52.8)	All (14)
AXO-AMP-FIS-TET	100	9	2	1	112 (48.5)	All (14)
AXO-AMP-TET-FIS-CHL	64	6	1	1	72 (31.2)	13
AXO-AMP-STR-FIS-CHL-TET	52	6	1	0	59 (25.5)	12
AXO-AMP-STR-FIS-CHL	48	6	0	1	55 (23.8)	11
AXO-AMP-STR-CIP	29	1	2	3	35 (15.2)	8
AXO-AMP-STR-AZI	20	0	0	2	22 (9.5)	6
AXO-AMP-FOX	20	1	0	0	21 (9.1%)	6
AXO-AMP-AUG2-FOX	16	0	0	1	17 (7.4)	6
AXO-AMP-STR-NAL-STX	19	0	0	0	19 (8.3)	6
AXO-AMP-STR-AZI-CIP	14	0	0	1	15 (6.5)	3

^a^Frequency and proportion of isolates with a given concurrent resistant pattern. ^b^Number of farms with the given concurrent resistance pattern. AXO, ceftriaxone; FOX, cefoxitin; AUG2, amoxicillin-clavulanic acid; AMP, ampicillin; AZI, azithromycin; CIP, ciprofloxacin; NAL, nalidixic acid; CHL, chloramphenicol; STR, streptomycin; TET, tetracycline; FIS, sulfisoxazole; STX, trimethoprim-sulfamethoxazole.

## 4 Discussion

In this study, all farms identified mastitis as the most frequent health problem of dairy cows and a primary driving factor for antibiotic use. This finding is consistent with other studies in the United States that also reported mastitis as the most common disease and the main reason for antibiotics use in dairy farms (8, 11, 46, 48, 49). Similar to previous studies in the United States (50, 51), most (86%, *n* = 14) of the farms in the present study used blanket dry cow therapy to manage mastitis. This practice may expose many animals and their gut commensal bacteria to antibiotics as portions of the administered dose or metabolites may enter the bloodstream and reach the gut (52).

Beta-lactam antibiotics such as cephalosporins (ceftiofur, cephapirin benzathine, and cephapirin sodium) and penicillin were the most frequently used antibiotics in the study farms. This finding concurs with a recent study by Nora et al. (12), who reported the consumption of large quantities of cephalosporins and penicillin in dairy cattle farms compared to other antibiotics. Similarly, the latest USDA survey reports (50, 53) and two other studies in the United States (8, 54) also showed that cephalosporins and penicillin are the most commonly used antibiotics to treat or prevent mastitis and other common diseases of dairy cattle. The previous studies demonstrated that frequent use of a given antibiotic leads to the emergence of resistance to antibiotics (47, 55–59). Regularly using cephalosporins, particularly ceftiofur, in the study farms is concerning as it could lead to the emergence of ESBL-producing bacteria such as *E. coli* (60).

ESBL-producing *E. coli* was detected from fecal samples of at least one animal in all 14 study farms resulting in 100% herd-level prevalence. This is higher than previous studies from the United States that reported 20% (5/25 farms) from Ohio (37), 85% (18/21) from Washington (30), and 4% (3/80) from Pennsylvania (61) dairy herds. However, this comparison should be interpreted with caution as these studies may differ in the criteria that they used to define a herd as positive (use of pooled manure samples vs. rectal fecal sample), sample processing steps (use or not using enrichment steps, type of media used for bacterial isolation) involved to determine the herd level status of ESBL-producing *E. coli*, which has different detection sensitivity and specificity. The widespread occurrence of ESBL-producing *E. coli* across the present study farms could be related to the frequent use of beta-lactam antibiotics, such as ceftiofur, that may subject them to selection pressure and other farm management related factors that favor the spread of the ESBL-producing *E. coli* or their ESBL genes.

The weighted within-herd prevalence of fecal ESBL-producing *E. coli* ranged from 8.0 to 100%, and the difference between within-herd prevalence was statistically significant across the farms. Detection of statistically significant variation in prevalence between different farms could be related to the differences in the type and level of beta-lactam antibiotics use and the presence of other influencing farm management related factors that were not within this study’s scope.

The within-herd prevalence of 8–100% in the present study is higher than a few previous studies conducted in the United States, including 3.3–100% (37) in Washington state and 0–33% in Ohio (33). Studies from other countries also reported a within-herd prevalence ranging from 5.2–86.7% (62, 63). However, it should be noted that these studies differ from the present study and each other in the microbiological techniques they used to isolate and identify the bacteria and the study farms’ selection criteria, which makes the comparison less plausible.

The prevalence of fecal ESBL-producing *E. coli* in the present study was 47.5% at an animal level, indicating a high level of colonization. The current prevalence report is higher than the previous report from Ohio dairy cattle (9.4%,70/747) (37) but closely similar to a report from European dairy cattle, 41% (37/90) (63). The fecal prevalence of ESBL-producing *E. coli* in this study is higher than the recently reported prevalence of 4.6% in United States cow-calf operations (64). This variation could be related to the differences in the type and frequency of beta-lactam antibiotics use and other managemental factors that may favor the occurrence and spread of ESBL-producing *E. coli* in the farms.

The fecal ESBL-producing *E. coli* prevalence in dairy calves was significantly (*p* < 0.001) higher than in cows (61.7% vs. 44%). This finding agrees with recent studies from North America (56, 61, 64–68) and Europe (63, 69–71) that reported a higher proportion of ESBL-producing or -third-generation cephalosporins (3GC) resistant-*E. coli* in calves compared to older cattle. Conclusive evidence as to why the higher prevalence of resistant bacteria in calves is not available and needs further study. It is hypothesized that feeding medicated milk replacers, colostrum, or milk from cows treated with beta-lactam antibiotics might have exerted selective pressure that led to the increased fecal carriage of ESBL-producing *E. coli* in calves (56, 63, 66, 69, 72). However, a recent study in the United States and Canada dairy calves showed a high prevalence of antimicrobial resistant *E. coli* in young calves that were not exposed to antimicrobials-containing milk replacers or did not feed milk from cows treated with antibiotics (66). Another alternative hypothesis for a higher prevalence of ESBL-producing *E. coli* in calves is related to the fitness of the resistant bacteria due to less bacterial diversity in the calves’ gut compared to adult dairy cattle with more diverse gut bacteria where competition is strong for ESBL-producing *E. coli* to thrive (71, 73, 74). A controlled study is needed to identify factors that promote a higher fecal ESBL-producing *E. coli* carriage in dairy calves than in adult cattle. Whatever the driving factor may be, this study suggests that calves may be an important source or reservoir of ESBL-producing *E. coli* and can be considered as sentinel animals for surveillance.

The detection of a higher proportion of ESBL-producing *E. coli* in manure is not surprising as manure contains a mixture of fecal samples, bedding, and wastewater pooled from different animal sources and has a high chance of containing the bacteria. Previous studies in the United States also reported a comparable level (53.5%; 62/116) of ESBL-producing *E. coli* from pooled manure samples (33). Most dairy farms in this study use manure as a fertilizer on pasture fields, which can contaminate the environment and water. Thus, the presence of ESBL-producing *E. coli* in the manure sample has significant public and environmental health implications. In our previous study, we detected a higher proportion of 3GC-resistant *E. coli* in dairy manure-amended soils compared to prairie soils not impacted by dairy cattle suggesting the need for proper management of manure to reduce the spread of resistant bacteria (16). The detection of ESBL-producing *E. coli* in feed (33.3%) and water (26%) samples in this study suggest that the bacteria may remain circulating in dairy farms through oral-fecal transmission via contaminated feed and water (75). Frequent cleaning of water and feed troughs may help to reduce the chance of maintenance of ESBL-producing *E. coli* in the dairy farm (5, 67).

This study also assessed animal-level factors that may influence the fecal prevalence of ESBL-producing *E. coli*. Recent use of ceftiofur (for treatment or prophylaxis), parity, and lactation status of the cow were significant predictors for the probability of ESBL-producing *E. coli* fecal carriage (*p* < 0.05). Cows recently receiving ceftiofur treatment (up to 6 months before sample collection) were 35% more likely (OR = 1.35) to carry ESBL-producing *E. coli* in their feces compared to cows not exposed to this antibiotic during the same period. Previous studies (4, 72, 76–79) also showed ceftiofur use leads to an increased probability of ESBL-producing *E. coli* detection or decreased susceptibility to 3GC, such as ceftriaxone in dairy cattle. Controlled studies in the United States (78) and Europe (80) showed a significant increase in ESBL mediating genes and mobile genetic elements (plasmids and prophages) in ceftiofur treated compared to non-treated dairy cows. In addition, a European study showed a restrictive use of ceftiofur and cefquinome (4th generation cephalosporin) in cattle significantly decreased the prevalence of fecal ESBL-producing *E. coli* in cattle (71).

Nevertheless, existing literature does not fully agree on the association between ceftiofur use and the probability of detection of *E. coli* with reduced susceptibility to 3GC. Some authors reported a lack of association between the two (37, 68), and others reported a decreased chance of recovering resistant *E. coli* following treatment with ceftiofur (72). However, most controlled and observational studies (37, 68) (including the present study) indicated a stronger association between ceftiofur use and the probability of detecting ESBL-producing *E. coli*. Thus, considering ways to reduce ceftiofur use (e.g., using only when sensitivity test result justifies) may help to reduce the ESBL-producing *E. coli* emergence and spread.

The likelihood of lactating cows having ESBL-producing *E. coli* in their feces were more than three times greater (OR = 3.42) than that of dry cows. The difference in the prevalence of fecal ESBL-producing *E. coli* may be related to the increased risk of mastitis and other diseases in lactating cows, prompting antibiotic use and favoring the emergence of ESBL-producing *E. coli* strains. This finding is significant because lactating cows are more likely to come into contact with humans than dry cows, and they also produce milk that can be contaminated. This increases the risk of transmission of ESBL-producing *E. coli* from cows to humans during the lactation period.

Cows with higher parity (≥3) were associated with an increased probability (OR = 1.33) of fecal ESBL-producing *E. coli* recovery compared to those cows with lower parity (≤2). This could be related to frequent exposure to beta-lactam antibiotics for managing mastitis and other diseases whose prevalence likely increases with age. Previous studies (81–83) showed a strong association between the prevalence or incidence of mastitis and parity (higher risk in older cows), which is also correlated with increased use of antibiotics that promote selection pressure on commensal *E. coli*.

Almost all (99.6%, 230/231) ESBL-producing *E. coli* tested for AST exhibited resistance to at least one of the 14 antimicrobial agents tested. The isolates displayed the highest level of resistance to ampicillin (99.1%, *n* = 231) and ceftriaxone (98.7%) which is expected as the isolates produce ESBL-enzymes that hydrolyze penicillin and extended-spectrum cephalosporins (14, 84). Three ESBL-producing *E. coli* isolates were identified as ESBL*-*producers by the selective chromogenic media and were unexpectedly susceptible to ceftriaxone, a 3GC. One isolate was sensitive to all the tested antimicrobial agents. This finding was unexpected as the selective media used in this study contains a supplement that is supposed to select isolates resistant to beta-lactam antibiotics such as penicillin and 3GC. This could be related to the reduced specificity of the screening media used to isolate ESBL-producing *E. coli* or later loss of expression of the ESBL gene (85).

A high prevalence of co-resistance was detected between ceftriaxone and older antibiotics such as ampicillin (97.8%, *n* = 231), tetracycline (79.7%), sulfisoxazole (59.7%), and streptomycin (54.5%). This resistance trend and prevalence are consistent with previous studies from the United States dairy cattle (16, 30, 61). Recently, Masse et al. (65) also reported a similar trend but a lower prevalence of co-resistance to these antibiotics in Canada. However, some authors used different initial screening steps (selective media) to isolate *E. coli*, making the comparison challenging. Some of these antibiotics (streptomycin and sulfisoxazole) were not mentioned by farmers as predominantly used antibiotics in their farms, suggesting the high level of resistance detected may not be related to their utilization. Further investigation is important to explain if these resistance phenotypes were co-selected with ESBL phenotypes, common resistance phenotypes reported in this study.

ESBL-producing *E. coli* exhibited low resistance to amoxicillin-clavulanic acid (8.7%, *n* = 231) and cefoxitin (10%). Generally, the growth of ESBL-producing *E. coli* is expected to be inhibited by both amoxicillin-clavulanic acid and cefoxitin, a cephamycin (14, 84, 86). As reported in other studies, the detection of resistance to these two antibiotics suggests the possible expression of multiple resistance phenotypes in the same *E. coli* isolates (5, 30, 65).

ESBL-producing *E. coli* displayed a higher level of concurrent resistance between ceftriaxone and other members of CIAs, such as streptomycin (55%), ciprofloxacin (18.6%), and azithromycin (13.4%). The prevalence of concurrent resistance is similar to a previous study from the United States (30) but 5 to 20 times higher than the reported prevalence from Canadian dairy farms (65). Other previous studies reported ESBL-producing *E. coli* often exhibit co-resistance to multiple CIAs (3, 68).

The overall prevalence of MDR ESBL-producing *E. coli* isolates in this study were 94.4% (218/231), suggesting an association between MDR and ESBL-phenotypes in *E. coli*. This finding is consistent with previous studies that reported a high prevalence of MDR phenotype1 in ESBL-producing *E. coli* isolates from cattle rectal fecal and farm manure samples (30, 64, 68). About 42.6% of MDR *E. coli* isolates in this study showed concurrent resistance to at least six classes of antibiotics, suggesting the ESBL-producing *E. coli* is a host of resistance genes which may complicate treatment if these bacteria cause infection or transfer the resistance genes to pathogenic bacteria. Multidrug resistant ESBL-*E. coli* was detected from all farms and all sample types, suggesting widespread occurrence across farms. Unlike recent studies from Canadian dairy farms (65), no difference was detected in the prevalence of the MDR across the five sample sources and study farms. The lack of detection of variation indicates that ESBL phenotype is a phenotypic marker of the MDR pattern in *E. coli,* irrespective of sample sources (5, 86, 87).

The study found two specific patterns of multidrug resistance ESBL-producing *E. coli*, AXO-AMP-STR-TET (52.8%), and AXO-AMP-FIS-TET (48.5%) were prevalent and detected in all sample types and study farms. The widespread occurrence of these patterns is a cause for concern regarding the spread of antibiotic resistance. The study calls for further research to understand the mechanisms behind the spread of these patterns, and the potential impact on animal and human health to identify strategies for reducing their prevalence in dairy farms.

The study found that 6.5% of ESBL-producing *E. coli* had concurrent resistance to at least one antibiotic in all CIAs tested in this study. This is a serious cause for concern as these antibiotics are of the highest importance for human health, and their efficacy could be compromised. However, concurrent resistance was detected from only three study farms, highlighting the need for continuous monitoring with targeted and localized interventions. Another study in the United States reported that CIAs such as macrolides and fluoroquinolones are commonly used to treat diseases in dairy calves and other non-lactating dairy cattle under 2 years of age, which could possibly drive the concurrent presence of resistance to these antibiotics (88). Other studies in humans reported the frequent use of 3GC as a risk factor for the co-occurrence of resistance to CIAs such as fluoroquinolones (89–91) which could be the case as two of these farms were frequently using ceftiofur, a 3GC.

## 5 Conclusion

This study showed a high prevalence of ESBL-producing *E. coli* in dairy cattle farms, with almost half of the tested animals positive for the bacteria. The prevalence of ESBL-producing *E. coli* varied significantly within each farm. The study found that recent treatment with third generation cephalosporins, cows with higher parity, and being calves was linked to an increased risk of fecal carriage of ESBL-producing *E. coli*. These findings suggest that interventions should target these identified variables. Almost all the ESBL-producing *E. coli* isolates were MDR with the highest resistance to ampicillin and ceftriaxone. Over 40% of ESBL-*E. coli* showed concurrent resistance to at least six classes of antibiotics. Concurrent resistance to ceftriaxone, ampicillin, tetracycline, sulfisoxazole, streptomycin, and chloramphenicol was high and widespread across all farms. It is important to indicate that the study farms and animals sampled from each farm were based on farmers that agreed to participate in the study and individual animals were selected based on convenience sampling. In addition, we acknowledge that the use of CHROMagar ESBL plates, while a widely accepted method, may result in some misclassifications. Despite these limitations, the findings of this study particularly the high prevalence of MDR ESBL-producing *E. coli* in the study area is significant and provides baseline data for future intervention measures. Further investigation, such as the longitudinal monitoring of ESBL-*E. coli* colonization in dairy cattle and possible transmission between cattle and farm workers who are in direct and frequent contact with animals and their feces is required.

## Data availability statement

The original contributions presented in the study are included in the article/Supplementary material, further inquiries can be directed to the corresponding author.

## Ethics statement

The animal study was approved by The University of Tennessee Institutional Animal Care and Use Committee (IACUC) registration number 2782-0722. The study was conducted in accordance with the local legislation and institutional requirements.

## Author contributions

BG: Conceptualization, Writing – review & editing, Formal analysis, Investigation, Methodology, Writing – original draft. AG: Investigation, Writing – review & editing. OK: Writing – review & editing, Conceptualization, Project administration, Supervision.

## Footnotes

^1^https://www.cdc.gov/narms/antibiotics-tested.html
