# Supplementary material for: Prevalence and antimicrobial resistance profiles of extended-spectrum beta-lactamase-producing Escherichia coli in East Tennessee dairy farms
Source: Front Vet Sci. 2023 Dec 18;10:1260433. doi: 10.3389/fvets.2023.1260433 (PMC10795760; doi:10.3389/fvets.2023.1260433)
Supplement: Supplementary file 1 [file Data_Sheet_1.docx]

**Supplementary Materials**

**Supplementary Table 1.** Antimicrobial agents (CMV4AGNF Panels), concentration range, and CLSI breakpoints for ESBL-*E. coli* isolates

| Antimicrobial Class (CLSI) | Antimicrobial Agent | Concentration (mg/mL) | Breakpoints (mg/mL) ^a^ | | |
| --- | --- | --- | --- | --- | --- |
|  |  |  | R | I | S |
| Beta-lactam/ Beta-lactamase inhibitor combinations | Amoxicillin/clavulanic acid | 1/0.5–32/16 | ≥ 32/16 | 16/18 | ≤ 8/4 |
| Penicillin | Ampicillin | 1 – 32 | ≥ 32 | 16 | ≤ 8 |
| Cephems | Cefoxitin | 0.5 – 32 | ≥ 32 | 16 | ≤ 8 |
| Cephalosporins | Ceftriaxone | 0.25 – 64 | ≥ 4 | 2 | ≤ 1 |
| Carbapenems | Meropenem | 0.06 – 4 | ≥ 4 | 2 | ≤ 1 |
| Macrolides | Azithromycin | 0.25 – 32 | ≥ 32 |  | ≤ 16 |
| Aminoglycosides | Gentamicin | 0.25 – 16 | ≥ 16 | 8 | ≤ 4 |
|  | Streptomycin | 2 – 64 | ≥ 32**^b^** |  |  |
| Quinolones | Ciprofloxacin | 0.015 – 4 | ≥ 1 | 0.5 | ≤ 0.25 |
|  | Nalidixic acid | 0.5 – 32 | ≥ 32 |  | ≤ 16 |
| Phenicols | Chloramphenicol | 2 – 32 | ≥ 32 | 16 | ≤ 8 |
| Folate pathway inhibitors | Sulfisoxazole **^c^** | 16 – 256 | ≥ 512 |  | ≤ 256 |
|  | Trimethoprim/sulfamethoxazole | 0.12/2.38 – 4/76 | ≥ 4/76 |  | ≤ 2,38 |
| Tetracyclines | Tetracycline | 4 – 32 | ≥ 16 | 8 | ≤ 4 |

**^a^** R: resistant; I: intermediate; S: susceptible. **^b^** The National Antimicrobial Resistance Monitoring System (NARMS) gram-negative Sensititre plate interpretive breakpoint was used for streptomycin. All other breakpoints presented in Table 1 were adopted from the CLSI M100-32 edition (46).

**Supplementary Table 2.** Prevalence of ESBL-*E. coli* in dairy cattle farms by sample type

| Sample type | Sample size (n)^a^ | No. Positive ^b^ | Prevalence (95% CI) |
| --- | --- | --- | --- |
| Rectal feces | 508 | 209 | 41.1% (36.8 - 45.6) |
| Manure | 30 | 14 | 46.7% (28.3 - 65.7) |
| Water | 19 | 5 | 26.3% (9.1- 51.2) |
| Feed | 15 | 5 | 33.3% (11.8 - 61.6) |
| Total | 572 | 233 | 40.7% (36.7- 44.9) |

CI: confidence interval; ^a^ Number of samples tested from each source.

^b^ Number of samples that yielded ESBL-*E. coli*

**Supplementary Table 3.** Prevalence of multidrug resistant ESBL-*E. coli* across various sample sources

| Sample Source | (No. of isolates) ^a^ | n (Prev. MDR  Isolates) ^b^ | n (Prev.) of resistance to ≥ 6 Antimicrobial Classes |
| --- | --- | --- | --- |
| Cows | 180 | 169 (93.9%) | 66 (36.7%) |
| Calves | 27 | 26(96.3%) | 15 (55.6%) |
| Manure | 14 | 14(100%) | 6 (42.9%) |
| Water | 5 | 5(100%) | 2 (40%) |
| Feed | 5 | 4 (80%) | 4 (80%) |
| Total | 231 | 218 (94.4%) | 93 (42.6%) |

^a^ Number of *E. coli* isolates from specific sources; No. MDR **^b^**: number and Prevalence of multidrug resistant *E. coli*; **^c^**n: frequency; Prev.: prevalence
